# Three-dimensional visualization of the microvasculature of bile duct ligation-induced liver fibrosis in rats by x-ray phase-contrast imaging computed tomography

**DOI:** 10.1038/srep11500

**Published:** 2015-07-27

**Authors:** Ruijiao Xuan, Xinyan Zhao, Doudou Hu, Jianbo Jian, Tailing Wang, Chunhong Hu

**Affiliations:** 1 College of Biomedical Engineering, Tianjin Medical University, Tianjin 300070, China; 2 Liver Research Center, Beijing Friendship Hospital, Capital Medical University, Beijing 100050, China; 3 Department of Pathology, China-Japan Friendship Hospital, Beijing 100029, China

## Abstract

X-ray phase-contrast imaging (PCI) can substantially enhance contrast, and is particularly useful in differentiating biological soft tissues with small density differences. Combined with computed tomography (CT), PCI-CT enables the acquisition of accurate microstructures inside biological samples. In this study, liver microvasculature was visualized without contrast agents *in vitro* with PCI-CT using liver fibrosis samples induced by bile duct ligation (BDL) in rats. The histological section examination confirmed the correspondence of CT images with the microvascular morphology of the samples. By means of the PCI-CT and three-dimensional (3D) visualization technique, 3D microvascular structures in samples from different stages of liver fibrosis were clearly revealed. Different types of blood vessels, including portal veins and hepatic veins, in addition to ductular proliferation and bile ducts, could be distinguished with good sensitivity, excellent specificity and excellent accuracy. The study showed that PCI-CT could assess the morphological changes in liver microvasculature that result from fibrosis and allow characterization of the anatomical and pathological features of the microvasculature. With further development of PCI-CT technique, it may become a novel noninvasive imaging technique for the auxiliary analysis of liver fibrosis.

Liver fibrosis is a reversible wound-healing response characterized by the accumulation of extracellular matrix. It occurs in almost all patients with chronic liver injury following diverse types of injurious intrusions, such as viral infection and alcoholic, drug or chemical toxicity^1^. Ultimately, liver fibrosis may lead to cirrhosis and reduce liver functional reserve^2^,^3^. The development and variation of liver fibrosis always correlates with changes in microvascular structure and morphology^4^,^5^,^6^,^7^. The microvascular changes are regarded as one of the most important and recently discovered pathophysiological features of liver fibrosis^8^,^9^. Microvasculature imaging in the liver can be employed to assess fibrosis progression and assist in the early finding of liver fibrosis and the objective evaluation of the degree of fibrosis. While the histologic assessment of a liver biopsy sample remains the gold standard for quantifying fibrosis, there is an increasing interest in the use of noninvasive methods to allow more frequent sampling and avoid the risks of percutaneous biopsy^1^. Imaging modalities can present the direct morphology and structure of liver vessels, and they can provide more detailed information about liver vessels or even replace biopsy. However, without contrast agents, the microvascular changes in the liver are invisible to the current imaging techniques due to the restrictions of their spatial resolution and contrast. Thus, there is an urgent need for new imaging modalities, and these techniques need to enable physicians to reconstruct accurate microvascular morphology of liver fibrosis and better understand pathologies.

X-ray phase-contrast imaging (PCI) is a promising imaging technique that can detect minute differences between biological soft tissues. It has approximately a 1000-fold higher sensitivity than conventional radiography^10^,^11^,^12^,^13^. PCI shows promise for improved biomedical imaging because it enables the production of images with high resolution and simultaneously requires lower radiation doses. Combined with computed tomography (CT), PCI-CT could exhibit the superior properties over conventional absorption-based CT, promising to provide high-resolution micro-CT images, especially for soft tissues. In recent years, it has been demonstrated that PCI-CT has outstanding potential to reveal detailed microstructures inside biological specimens^14^,^15^,^16^,^17^,^18^,^19^,^20^,^21^,^22^,^23^,^24^,^25^. In imaging blood vessels, previous studies have shown that blood vessels down to the micrometer level can be clearly revealed by PCI-CT without iodine contrast agents^7^,^26^,^27^,^28^,^29^,^30^,^31^. By means of the PCI-CT and three-dimensional (3D) visualization technique, high-resolution 3D visualizations of microvascular networks are presented, and the technique provides new possibilities for analyzing and characterizing anatomical and pathological features of the vessels, especially for differentiating between healthy and diseased vessels.

In liver fibrosis induced by bile duct ligation (BDL), vascular lesions and bile duct lesions influence each other, and they promote the progression of the liver disease together. The effective visualization of vascular and bile ductular structures facilitates the study of microcirculation mechanisms and pathological changes associated with liver fibrosis induced by BDL. However, in the study of liver imaging, differentiating bile ducts and blood vessels is still a challenging work without using a contrast agent. In this study, liver fibrosis induced by BDL in rat was used to perform the experiment without contrast agents, and the microvasculature from different stages of liver fibrosis samples were visualized using PCI-CT. The purpose of this study was to explore the potential of PCI-CT in microvasculature imaging for the BDL-induced liver fibrosis model and to determine whether different types of blood vessels can be differentiated based on 3D microvascular structures of rat livers.

## Results

### PCI projection images

**Figure 1:**
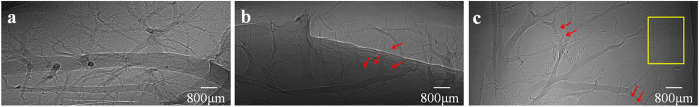
The projection images of the livers. Images are obtained from (**a**) group A, (**b**) group B, and (**c**) group C, and they characterize a normal, mild and severe liver fibrosis sample, respectively. The red arrows indicate the bile ducts accompanying the portal veins in (**b**) and (**c**). In (**c**), the ductular proliferation is shown within the yellow rectangular region.

The projection images of the livers were shown in Fig. 1. The liver vessels were visualized to a scale of tens of micrometers without contrast agents. The architecture of the vascular network was clearly revealed on the image, and the vessels with a minimum diameter of approximately 50 μm were detected. The normal liver vessels appeared ordered and regular, and the vascular branches were gradually thinned (Fig. 1a). The portal veins were normally accompanied with bile ducts, and the bile ducts can be visualized in both mild (Fig. 1b) and severe (Fig. 1c) liver fibrosis samples. In the severe liver fibrosis samples, some rough granular structures, as shown in the area of yellow rectangular region, were presented. They were demonstrated to be the result of ductular proliferation according to pathobiology, as described in the following sections.

### PCI-CT images and pathological sections

**Figure 2:**
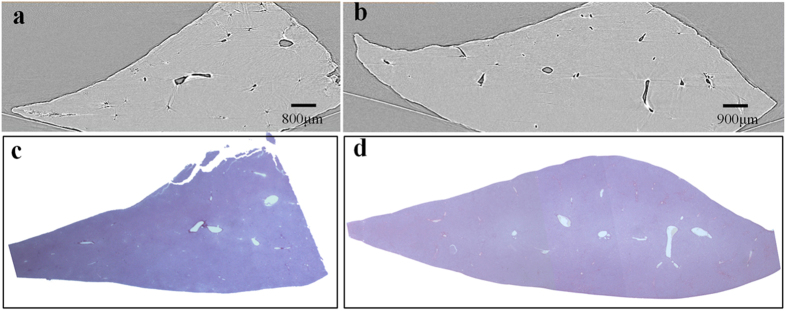
Histological sections and CT images of the normal and mild liver samples. The PCI-CT images in (**a**) normal and (**b**) mild liver fibrosis samples are presented, and the corresponding histological sections are shown in (**c**) and (**d**). The magnification of histological section is 12.5×.

**Figure 3:**
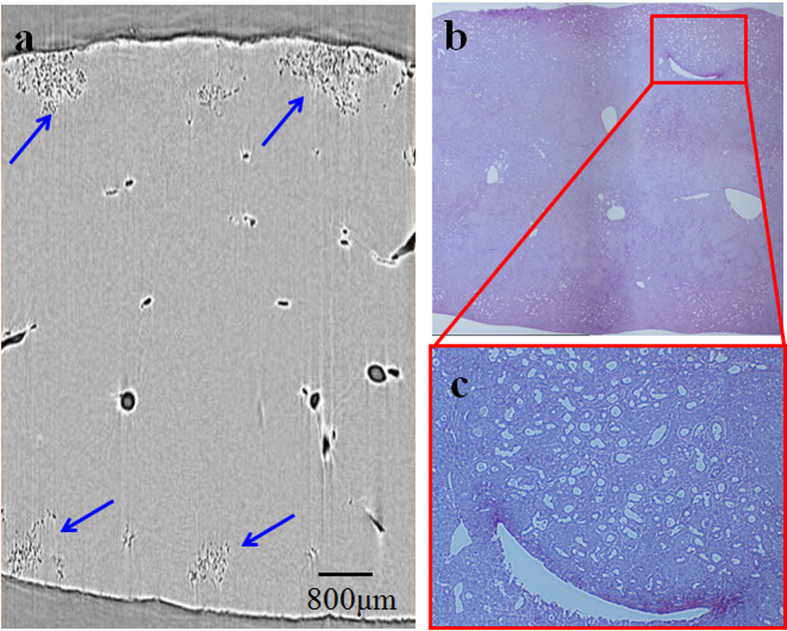
CT image and histological section of the severe liver fibrosis sample. (**a**) One slice of PCI-CT reconstruction images. The ductular proliferations are marked by blue arrows. (**b**) Histological section. (**c**) The amplified images surrounded by the red rectangle regions. In (**b**) and (**c**), magnification is 40× and 100×, respectively.

The PCI-CT images and corresponding histological sections were shown for different stages of liver fibrosis (Fig. 2, Fig. 3). Basically, the CT image had a close resemblance to the histological section, and it could obtain information on the actual structures seen in histopathologic analysis, which highlighted the high degree of sensitivity of PCI-CT. In the severe liver fibrosis sample, abundant ductular proliferations could be clearly observed near the edge of the liver surface and presented cluster distributions in shape (Fig. 3a, Fig. 3b). The structural details of the ductular proliferation were revealed by the amplified image of the red rectangle region in Fig. 3b (Fig. 3c).

### 3D visualization of the microvasculature

**Figure 4:**
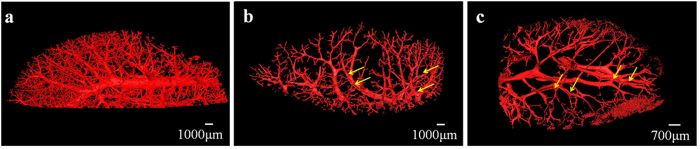
3D microvasculature rendering from different stages of liver fibrosis samples. The microvascular structures in (**a**) normal, (**b**) mild and (**c**) severe liver fibrosis samples are presented. As marked by the yellow arrows, the bile ducts accompanied by the portal veins could be observed in (**b**) and (**c**). In (**c**), the ductular proliferation marked by blue arrows is revealed at the end of vessels.

**Figure 5:**
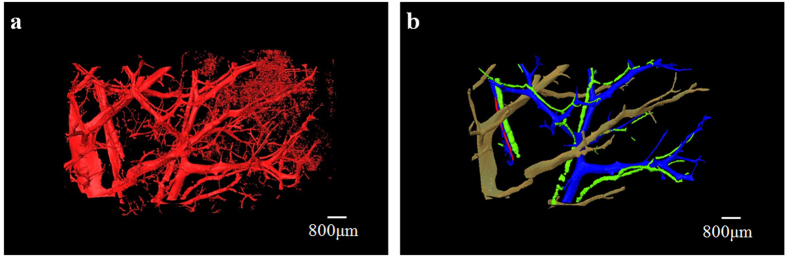
The segmentation of different types of blood vessels. To better differentiate blood vessels, 3D microvascular structures of the same sample as Fig. 5c is shown in (**a**) based on another view. As shown in (**b**), portal veins (blue), hepatic veins (yellow), arteries (red) and bile ducts (green) are segmented and marked by use of different colors when omitting the ductular proliferation.

Although the PCI projection images can clearly display microvascular structures of the livers, superimposed vessel structures cannot be differentiated. 3D microvascular structures from the different stages of liver fibrosis samples were reconstructed by combining PCI-CT images with 3D visualization technology (Fig. 4). Figure 4a clearly exhibited the vascular tree of the normal liver sample, and the 3D microvascular structure was presented with regular shapes and stereoscopic effects. In the liver fibrosis samples, vessel deformations, such as vessel disorders and abnormal vessel morphology, became apparent due to compression caused by fibrosis tissues (Fig. 4b and Fig. 4c). In addition, the irregular vessel diameter changes and tortuous vessels became more apparent as the fibrosis stage increased. The bile ducts, which were marked by the yellow arrows in Fig. 4b and Fig. 4c, were clearly presented because the bile ducts expanded with the ligation operation. The ductular proliferation, as marked by the blue arrows, was also reconstructed in the severe liver fibrosis sample (Fig. 4c). Figure 5a showed 3D microvascular structures of the same sample as Fig. 4c from another view. To differentiate the different types of blood vessels and bile ducts without considering ductular proliferation, the vessels in Fig. 5a were separately segmented and represented using different colors, as shown in Fig. 5b. Obviously, the large vessels accompanied by bile ducts (green) were portal veins (blue), and the vessels without the accompanying bile ducts were demonstrated to be hepatic veins (pale yellow). Additionally, another small vessel accompanied by a portal vein may be the artery (red). The associated animation in supplementary material (Video S1) clearly describes 3D microvascular structures of the severe liver fibrosis sample.

### Sensitivity, specificity, and accuracy of PCI-CT

3D microvasculatures were reconstructed from three mild liver fibrosis samples and three severe liver fibrosis samples. 190 regions of interest (ROIs) were selected from the obtained 3D microvasculatures, each ROI consisting of 200*200*200 voxels. The ten ROIs were used to train radiologists, and they were not included in the following analysis. Therefore, a total number of 180 ROIs in six samples were available for analysis.

Histological sections showed the presence of bile duct in 153 of 180 ROIs (85.0%). Portal vein and hepatic vein were detected in 128 ROIs (71.1%) and 132 ROIs (73.3%), respectively. Ductular proliferation was found in 45 ROIs (25.0%). According to 3D microvascular structure characteristics in liver fibrosis samples, different types of blood vessels and bile ducts were detected with good sensitivity (all, >90%), excellent specificity (all, >80%) and excellent accuracy (all, >90%), resulting in Cohen k values from 0.78 to 0.94 for all types of blood vessels and bile ducts (Table 1). Interreader agreement analysis resulted in a k value of 0.84 for bile duct, 0.82 for portal vein, 0.82 for hepatic vein, and 0.91 for ductular proliferation (all *P* <0.001).

## Discussion

Our present study demonstrates that PCI-CT can substantially improve the radiographic contrast of liver tissues *in vitro* without any contrast agents and clearly reveal 3D microvasculature structure in liver fibrosis of rats induced by BDL. Different types of blood vessels (portal veins and hepatic veins), ductular proliferation and bile ducts, can be distinguished with good sensitivity, excellent specificity, excellent accuracy, and good interreader agreement based on 3D microvascular structure characteristics in liver fibrosis samples. Ductular proliferation, which presented cluster distributions near the edge of the liver surface, is detected in the severe liver fibrosis sample. A comparison between the CT images and the histological section of the sample shows that PCI-CT depicts microvascular anatomy details that could otherwise be seen only in histopathology.

By means of PCI technique, a study of liver fibrosis imaging has been developed by the investigators; however, these studies were performed in two dimensions and suffered from the overlapping of structures^4^,^5^,^6^. Our previous study has demonstrated that PCI-CT can present an accurate 3D morphology of the microvasculature based on liver fibrosis model in rats induced by human albumin and can characterize different stages of fibrosis progression based on high-resolution 3D vessel images^7^. Liver fibrosis induced by BDL represents an experimental model of human chronic biliary fibrosis. In the previous studies, differentiating bile ducts from blood vessels proved to be difficult^32^. In our study, accurate 3D microvasculature structures are presented, and different types of vessels are distinctly distinguished. Furthermore, the microvascular structures change as fibrosis develops further. Thus, the PCI-CT technique facilitates the study of microcirculation mechanisms and pathological changes to the microvasculature in liver fibrosis induced by BDL. It can provide useful information for the diagnosis of liver fibrosis, and the correlation between the liver fibrosis degree and vessel structure could allow for fibrosis staging.

While great progress has been made in our study, several limitations remain. First, the isolated animal samples are the main subjects in our experiments, and this *in vitro* study aims to explore the potential for future *in vivo* use. In principle, PCI-CT can be performed for *in vivo* imaging with acceptable doses by using optimized design of experimental instrument and new reconstruction CT algorithms^22^,^23^,^24^,^33^,^34^. In recent years, *in vivo* biomedical studies of PCI and PCI-CT have covered a wide range of pathologies and organs^21^,^35^,^36^,^37^,^38^. Next work, the study will be performed *in vivo* to further investigate the PCI-CT value in hepatic fibrosis analysis. Second, the CCD spatial resolution of our study is 9 μm, which is not adequate for accurate microstructures observation of ductular proliferation. The theoretical spatial resolution of PCI can reach the submicron level, and further increase in spatial resolution can solve this issue. Third, the small field-of-view in PCI is not suited for imaging large samples; however, a recent study shows that PCI-CT can allow the visualization of breast tissues structures and abnormalities in a large breast sample^24^,^39^. Finally, current PCI techniques are mostly based on synchrotron radiation, which hinders its potential clinical application. Recently, PCI techniques on a conventional X-ray tube have been developed, which will be an important step toward its clinical implementation^40^,^41^,^42^.

In summary, PCI-CT can clearly depict the morphology of the microvasculature from different stages of liver fibrosis induced by BDL without contrast agents and better characterize anatomical properties and pathological features of the microvasculature. Furthermore, this technique can differentiate between types of blood vessels and bile ducts. With further development of the PCI-CT technique, it may become a novel *in vivo* imaging tool for imaging the microvasculature in liver fibrosis models, which will provide a sensitive noninvasive imaging technique for auxiliary diagnosis and analysis of liver fibrosis.

## Methods

### Sample preparation

All the procedures involving in animal experiments were reviewed and approved by the Research Ethics Committee of Beijing Friendship Hospital, Capital Medical University (Permit Number: 12-1004). The methods were carried out in accordance with the approved guidelines. Male adult Sprague-Dawley rats, weighing 180–220 g, were used in the study. They were fed on a standard laboratory diet with free access to water and were housed in isolated cages with a 12-hour light-dark cycle. Nine rats were randomly divided into three groups: control group (group A), mild liver fibrosis (group B) and severe liver fibrosis (group C). The control group (3 rats) had a sham operation performed, in which the common bile ducts were exposed and manipulated without ligation. In group B (3 rats) and group C (3 rats), the ligation of the common bile duct (CBD) was conducted to induce biliary cirrhosis. Briefly, under phenobarbital anesthesia, the CBD was double-ligated using 4.0 silk after a midline abdominal incision. Rat livers were excised 10 days (group B) or 30 days (group C) after ligation of the CBD. All the liver samples were fixed in 4% formaldehyde solution for further experiments.

### Image acquisition

**Figure 6:**
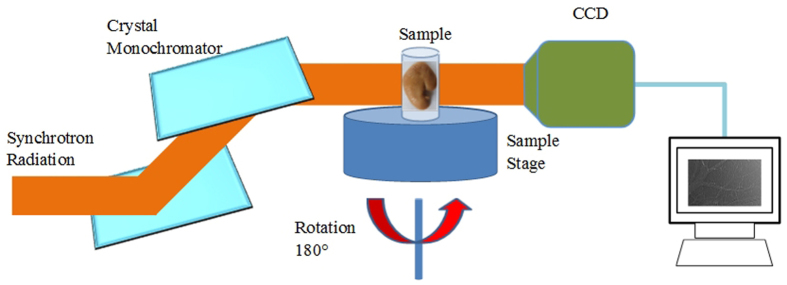
Schematic diagram of the PCI experimental setup at BL13W1 in SSRF. A monochromatized synchrotron radiation x-ray is projected on a sample mounted on a rotating sample stage, and the transmitted beam is recorded by an image detector after the x-ray propagates a proper distance from the object. For tomographic scans, the sample can be rotated within 180° to produce the projection images at different views.

The experiments were conducted at x-ray imaging and biomedical application beamline (BL13W1) of Shanghai Synchrotron Radiation Facility (SSRF) in China. X-rays were produced from a 3.5 GeV electron storage ring, and the tunable energy range of the x-ray beam was 8–72.5 keV. The beam was monochromatized via a double silicon crystal monochromator with Si(111) and Si(311) crystals. The samples were placed 34 m from the source, and the distance between the sample and the detector could be adjusted from 0 cm to 8 m. A thin (100 μm) CdWO_4_ cleaved single-crystal scintillator and a charge coupled device (CCD) camera were used to obtain images. A schematic of the experimental setup was shown in Fig. 6. In the PCI imaging system, the incident white synchrotron x-ray beam emerging from the accelerator was monochromatized by a double-crystal monochromator. By setting a proper sample-detector distance, the object was illuminated by the highly parallel and monochromatic x-ray beam, and the transmitted beam was measured by a detector.

The x-ray beam energy in the experiments was adjusted to 24 keV, and the distance between the samples and the CCD was 1.2 m. Projection images were collected by an x-ray CCD with a pixel size of 9 μm × 9 μm, and the exposure time per projection image was 12 ms. During the CT data acquisition, a total of 1200 projection images were collected when the samples were rotated within 180°. In addition, 10 dark images (dark signal in the absence of photons) and 20 flat images (with no sample in the beam) were used to perform a dark-field correction and a flat-field correction, respectively^43^. Projection images were then utilized to reconstruct the CT images using the filtered back projection (FBP) algorithm. Finally, the 3D reconstructions were performed using Amira (Visage Imaging, Berlin, Germany) and the 3D morphology of the microvasculature could be observed from different views.

### Histological analysis

After imaging studies, liver samples were embedded in paraffin, cut into 4-μm sections, stained with Sirius Red and observed with an optical microscope. The histological section and its corresponding analysis were performed by an experienced pathologist (T.W., with more than 50 years of experience in liver pathology). Histological findings served as the reference standard for interpretation of the CT images of the samples.

### Evaluation of the potential of PCI-CT

Combined with CT images and corresponding histological sections, 3D microvascular structures in the liver fibrosis samples were analyzed, and the characteristics of blood vessels, including portal vein, hepatic vein, ductular proliferation and bile duct, were established. A method as described in ref. 23 was used to analyze sensitivity, specificity, and accuracy of PCI-CT for the detection of the portal vein, hepatic vein, ductular proliferation and bile duct. As mentioned above, the ten ROIs of 3D microvasculatures in liver fibrosis samples, were used to train radiologists (C.H. and X.Z., with more than 8 years of experience in liver imaging), and two radiologists were blinded to histopathologic findings. By applying the established 3D microvascular structure characteristics, the presence or absence of the portal vein, hepatic vein, ductular proliferation and bile duct was assessed independently by both readers. In cases of disagreement, a consensus was reached through discussion.

### Statistical analyses

Statistical analyses were performed by one author (R.X.) with SPSS software (version 20; IBM, Chicago, USA). Cohen k was calculated to determine agreement between PCI-CT and histological findings and interreader agreement. A *P* value of less than 0.05 was considered to indicate a statistically significant difference.

## Additional Information

**How to cite this article**: Xuan, R. *et al.* Three-dimensional visualization of the microvasculature of bile duct ligation-induced liver fibrosis in rats by x-ray phase-contrast imaging computed tomography. *Sci. Rep.* **5**, 11500; doi: 10.1038/srep11500 (2015).

## Supplementary Material

Supplementary Information

Supplementary Video 1

## Figures and Tables

**Table 1:** Statistical data for sensitivity, specificity, and accuracy of PCI-CT in the detection of bile duct, portal vein, hepatic vein, and ductular proliferation.

	Prevalence*	**Sensitivity (%)**†	**Specificity (%)**†	**Accuracy (%)**†	**k Value**
Bile duct	153 (85)	94.1 (89.1,97.3)	92.6 (75.7,99.1)	93.9(90.4,97.4)	0.78
Portal vein	128 (71.1)	97.7 (93.3,99.5)	84.6 (71.9,93.1)	93.9(90.4,97.4)	0.85
Hepatic vein	132 (73.3)	97.7 (93.5,99.5)	81.3 (67.4,91.1)	92.8(89.0,96.6)	0.82
Ductular proliferation	45 (25)	93.3 (81.7,98.6)	99.3 (95.9,99.9)	98.3(96.5,99.9)	0.94

Note: Data are based on 180 ROIs. For all comparisons, *P* was less than 0.001.

^*^Prevalence was determined according to histological findings. Data are numbers of ROIs. Numbers in parentheses are percentages.

^†^Numbers in parentheses are the 95% confidence intervals.
