# Supplementary material for: Three-dimensional visualization of the microvasculature of bile duct ligation-induced liver fibrosis in rats by x-ray phase-contrast imaging computed tomography
Source: Sci Rep. 2015 Jul 27;5:11500. doi: 10.1038/srep11500 (PMC4515745; doi:10.1038/srep11500)
Supplement: Supplementary Information [file srep11500-s1.pdf]

Three-dimensional visualization of the microvasculature of bile duct ligation-induced liver fibrosis in rats by x-ray phase-contrast imaging computed tomography

Ruijiao Xuan [1*], Xinyan Zhao [2*], Chunhong Hu [1], Doudou Hu [2], Jianbo Jian[1], Tailing Wang[3]

[1]College of Biomedical Engineering, Tianjin Medical University, Tianjin 300070, China, [2]Liver Research Center, Beijing Friendship Hospital, Capital Medical University, Beijing 100050, China, [3]Department of Pathology, China-Japan Friendship Hospital, Beijing 100029, China.

Supporting information legends

Video S1. Animated view of the 3D microvascular structures of the severe liver fibrosis sample. This is the same 3D model shown in Fig.6. The rotation of the model permits the viewers to better observe microvascular structures, and differentiate the different types of blood vessels.
